# Interaction of carbonic anhydrase I released from red blood cells with human plasma in vitro

**DOI:** 10.1093/mtomcs/mfae028

**Published:** 2024-05-29

**Authors:** Maryam Doroudian, Jürgen Gailer

**Affiliations:** Department of Chemistry, University of Calgary, 2500 University Drive NW, Calgary, AB T2N 1N4, Canada; Department of Chemistry, University of Calgary, 2500 University Drive NW, Calgary, AB T2N 1N4, Canada

**Keywords:** Red blood cell lysis, Zn-metalloprotein, Plasma protein binding, Endothelium, Toxic metals, Mechanism of toxicity

## Abstract

Red blood cells (RBCs) constitute ∼50% of the bloodstream and represent an important target for environmental pollutants and bacterial/viral infections, which can result in their rupture. In addition, diseases such as sickle cell anaemia and paroxysmal nocturnal haemoglobinuria can also result in the rupture of RBCs, which can be potentially life-threatening. With regard to the release of cytosolic metalloproteins from RBCs into the blood-organ system, the biochemical fate of haemoglobin is rather well understood, while comparatively little is known about another highly abundant Zn-metalloprotein, carbonic anhydrase (CA I). To gain insight into the interaction of CA I with human blood plasma constituents, we have employed a metallomics tool comprised of size-exclusion chromatography (SEC) coupled online with an inductively coupled plasma atomic emission spectrometer (ICP-AES), which allows to simultaneously observe all Cu, Fe, and Zn-metalloproteins. After the addition of CA I to human blood plasma incubated at 37°C, the SEC-ICP-AES analysis using phosphate buffered saline (pH 7.4) after 5 min, 1 h, and 2 h revealed that CA I eluted after all endogenous Zn-metalloproteins in the 30 kDa range. Matrix-assisted laser desorption-time of flight mass spectrometry analysis of the collected Zn-peak confirmed that CA I eluted from the column intact. Our *in vitro* results suggest that CA I released from RBCs to plasma remains free and may be actively involved in health-relevant adverse processes that unfold at the bloodstream-endothelial interface, including atherosclerosis and vision loss.

## Graphical Abstract

**Graphical Abstract:**
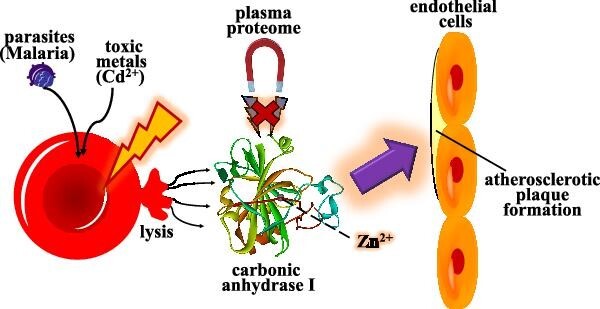
Toxic metal-induced red blood cell microbursts liberate carbonic anhydrase I which then impinges on endothelial cells thus possibly contributing to the etiology of atherosclerosis.

## Introduction

Carbonic anhydrases (CA) represent a family of ∼15 Zn-metalloenzymes that play a pivotal role in health and disease as they are involved in several physiological processes, including pH regulation, respiration, electrolyte transport, metabolism, bone resorption, and calcification.^1,2^ While CA IX and XII are cell membrane proteins that are expressed in various tissues and hold potential as biomarkers for certain cancers,^1,3^ the CA present in red blood cells (RBCs) is a monomeric ∼30 kDa Zn-metalloprotein that is primarily responsible for mediating the transport of the waste product CO_2_ from internal organs to the lungs.^4^ While human RBCs contain at least 1587 cytosolic proteins,^5^ the most abundant protein therein is haemoglobin (Hb, ∼5.5 mM), followed by the second most abundant protein CA I,^6^ which is present at concentrations of ∼12 mg/g of Hb.

The average lifetime of RBCs in the human bloodstream is approximately 120 days. However, a variety of events can significantly reduce their longevity due to their lysis, which will cause haemolytic anaemia. When the extent of RBC lysis is above a certain threshold, it can result in chronic health issues, while their sudden rupture can result in potentially life-threatening conditions including ischaemic stroke.^7^ In terms of events that can contribute to RBC lysis, one needs to consider the infection of the host with bacteria, viruses, and/or parasites (e.g. malaria^8^), the exposure of the host to toxic chemicals [e.g. arsine (AsH_3_)^9^], ageing^10^ as well as diseases, including sickle cell anaemia,^11^ hereditary spherocytosis,^12^ and/or paroxysmal nocturnal haemoglobinuria,^13^ with the latter involving the rupture of RBCs while patients are asleep.

The sudden release of Hb from ruptured RBCs into the blood plasma can result in kidney failure if the binding capacity of the plasma protein haptoglobin (Hp)—which tightly binds to Hb in the blood plasma to form a Hp-Hb complex—is overwhelmed.^14^ The fact that Hp-Hb complexes have been detected in the blood plasma of multiple sclerosis patients, stroke patients,^7^ and healthy controls implies that the rupture of RBCs does not appear to adversely affect human health as long as the continuous removal of cell debris, including Hb from the bloodstream is not compromised. Interestingly, not much is known about the fate of another abundant RBC metalloprotein, namely CA I, once it is released into plasma following a lysis event. Gaining insight into the fate of CA I in the biologically complex blood plasma, which contains >5000 plasma proteins,^15^ however, is exceedingly challenging, unless appropriate analytical methods are employed. In this regard, metallomics techniques have inherent potential to achieve this goal,^16^ as the analysis of plasma for a sub-proteome, such as the plasma metalloproteome, allows to considerably reduce the analytical complexity of analysing plasma for all contained proteins.^17^ Previous studies have revealed that a metallomics technique based on size-exclusion chromatography (SEC) coupled on-line to an inductively coupled plasma atomic emission spectrometer (ICP-AES) can conclusively distinguish between the absence of a binding event of a small molecular weight and the non-toxic arsenic compound arsenobetaine (AB)^18^ to plasma proteins from the binding of the toxic metal ion Cd^2+^ to distinct plasma proteins in blood plasma.^16^

To better understand the biomolecular events that unfold after the rupture of RBCs in the bloodstream, we sought to investigate the biomolecular fate of the Zn metalloprotein of interest in plasma. To this end, the previously developed SEC-ICP-AES technique was utilized to probe the interaction of bovine CA in rabbit and CA I in human blood plasma.^19^ Importantly, the use of phosphate buffered saline (PBS-buffer) is absolutely critical when blood plasma is to be analysed for the contained Cu, Fe, and Zn-metalloproteins as previous research has demonstrated that the utilization of other biochemical buffers, such as MOPS, HEPES, and Tris buffer will result in the mobilization of iron and zinc from their respective binding sites on their respective metalloproteins, therefore resulting in artifacts.^20^ Given the capability of SEC-ICP-AES to selectively and simultaneously monitor the major Cu, Fe, and Zn-metalloproteins contained in blood plasma, it was deemed to be well suited to unravel the biomolecular fate of CA I after it is released from RBCs to blood plasma by spiking the latter with pure Zn metalloprotein. While the well-established stability of CA I^21^ is expected to result in the detection of an additional peak in the Zn-specific chromatogram, its retention time will provide important insight into the interaction of this Zn-metalloprotein with other plasma proteins (e.g. presence of a CA I binding protein) at near physiological conditions.

## Experimental

### Chemicals, materials, and solutions

Phosphate buffered saline powder sachets (PBS, 0.01 M NaH_2_PO_4_, 0.138 M NaCl, 0.0027 M KCl, pH 7.4), CA from bovine erythrocytes (≥2000 W-A units/mg protein), and CA I from human erythrocytes (100–500 W-A units/mg protein) were obtained from Sigma-Aldrich (St. Louis, MO, USA). A mixture of gel filtration standards (bovine thyroglobulin—670 kDa, bovine γ-globulin—158 kDa, chicken ovalbumin—44 kDa, horse myoglobulin—17.5 kDa, and vitamin B_12_—1.35 kDa) was purchased from Bio-Rad Laboratories (Hercules, CA, USA) and reconstituted as specified. PBS-buffer was prepared by dissolving one sachet in deionized water obtained from a Simplicity UV water purification system (Millipore, Billerica, MA, USA) before being adjusted to pH 7.4 with 4.0 M HCl/NaOH and filling to 1 L. All pH measurements were performed with a VWR Symphony SB20 pH meter (Thermo Electron Corporation, Beverly, MA, USA). The PBS-buffer mobile phase was filtered through a 0.45 µm pore size filter (Mandel Scientific, Guelph, ON, Canada) and sparged with N_2_ for the duration of the experiment to reduce the amount of dissolved oxygen.

### Sample preparation

New Zealand white rabbit plasma (sodium heparin, pooled, unspecified gender) was purchased from BIOIVT (Westbury, NY, USA) and kept in liquid nitrogen. To generate human plasma, blood was collected from a volunteer following a protocol approved by the Calgary Conjoint Health Ethics Board (ethics approval REB15-1138-REN9). Plasma was prepared as previously described^22^ and stored in liquid nitrogen before use.

Individual vials of both rabbit and human plasma were thawed at room temperature (20°C) for 45 min and then filtered through 0.45 µm PVDF low protein-binding Millex syringe-driven filters (Merck Millipore Ltd, Tullagreen, Co. Cork, Ireland). Then 500 μL of plasma were transferred into cryovials and incubated at 37°C for 30 min. Thereafter, 460 µL of PBS buffer was added, followed by the addition of 40 μL of freshly prepared bovine CA solution (0.197 mM) for rabbit plasma and human CA I solution (0.167 mM) for human plasma, followed by mixing. Aliquots (0.5 mL) of either spiked rabbit plasma (containing 515 ng of Zn in the form of CA) or spiked human plasma (containing 437 ng of Zn in the form of CA) were removed after 5 min, 1 h, and 2 h and injected into the SEC-ICP-AES system. All experiments were conducted in triplicate.

### Instrumentation

The employed SEC-ICP-AES system was comprised of an Agilent 1200 series binary pump SL HPLC pump followed by a Rheodyne 9010 injector equipped with a 500 µL sample loop. A prepacked Superdex 200 Increase 10/300GL (30 × 1.0 cm ID, 8 µm particle size) high-resolution SEC column was utilized at a flow rate of 0.75 L min^−1^ using a PBS-buffer mobile phase. Simultaneous multi-element specific detection of C (193.091 nm), Cu (324.754 nm), Fe (259.940 nm), Zn (213.856 nm), and S (180.731 nm) in the column effluent was achieved with the Prodigy, high-dispersion, radial-view ICP-AES (Teledyne Leeman Labs, Hudson, NH, USA) with the following parameters: a radio frequency (RF) power of 1.3 kW, an Ar gas coolant flow rate of 19 L min^−1^, auxiliary flow rate of 0.5 L min^−1^, and a nebulizer gas pressure of 25 psi. A 240-s delay was implemented between injection and the start of data acquisition, where data were collected for the following 660 s. The raw data collected using the ICP-AES data acquisition and controller software (SALSA) were imported into SigmaPlot 15 and smoothed using the bisquare algorithm. Peak areas and retention times were determined using Origin software (Version 2022).

### Mass spectrometry

Using 50 mM Tris buffer pH 7.4, SEC column fractions that contained the presumed CA I were collected and submitted for analysis by matrix-assisted laser desorption ionization—time of flight mass spectrometry (MALDI-TOF MS). An AB Sciex Voyager Elite MALDI TOF instrument was used in linear mode to analyse the samples. Employing a two-layer sample preparation method, a 10× dilution of the sample was achieved by incorporating 0.1% TFA in water. The initial layer consisted of 0.6 µL of 10 mg/mL sinapinic acid dissolved in 80% acetone and 20% methanol, followed by a drying period. The subsequent layer comprised a 1:1 mixture of the sample solution and 10 mg/mL sinapinic acid dissolved in a 1:1 acetonitrile/water solution with 0.1% TFA. Subsequently, 1 µL of the second layer mixture was spotted onto the first layer.

## Results and discussion

The chronic exposure of certain human populations to a variety of environmental pollutants is associated with the development of a variety of diseases,^23^ but the underlying biomolecular processes remain only partially understood. For instance, the chronic exposure of humans (i.e. taxi drivers) to the toxic metal Cd^2+^ has been shown to be associated with RBC fragility,^24^ but the detailed biomolecular events that result in this condition require much more detailed insight to better define the associated exposure-response relationship. The fact that humans are simultaneously exposed to multiple pollutants^25^ and that the synergistic effects of individual pollutants^26^ may adversely affect the stability of RBCs underscores the pressing need for further research pertaining to the biomolecular interactions of multiple pollutants within the bloodstream-organ system. While the biochemical fate of the Fe-metalloprotein Hb that unfolds after it is released from RBCs into the bloodstream-organ system is fairly well understood,^27^ comparatively less is known about the fate of the Zn-metalloprotein CA once it is liberated from RBCs. In an attempt to overcome the inherent difficulty that is associated with observing the interaction of this Zn-metalloprotein with thousands of relevant biomolecules in blood plasma, a developed metallomics tool was deemed to be uniquely suited to be capable of providing new insight into these interactions.^16,28^

The control experiments involved the incubation of rabbit plasma and human blood plasma at 37°C, followed by the analysis of sample aliquots by SEC-ICP-AES after 5 min, 1 h, and 2 h. To visualize the profiles of the eluting plasma proteins, carbon-specific chromatograms were overlaid for all time points, which revealed four broad C-peaks, each corresponding to hundreds of proteins. Importantly, these ‘protein profiles’ remained largely consistent for both rabbit (ESI 1) and human (ESI 2) plasma over the 2-h period, demonstrating the reproducibility of the employed metallomics technique. The simultaneously observed Cu, Fe, and Zn-metalloproteome profiles for both human and rabbit plasma were in good agreement in terms of the number and relative intensity of metal peaks compared to those observed in previous studies,^17,18^ and also revealed that the integrity of all endogenous metalloproteins was not compromised over this time period. Furthermore, the high reproducibility of the Cu, Fe, and Zn metalloproteome obtained over the 2-h period represents a valuable and unique endogenous analyte ‘metal signature’ for both rabbit and human plasma, which validates the results obtained after spiking these fluids with pure bovine CA and CA I, respectively.

**Fig. 1:**
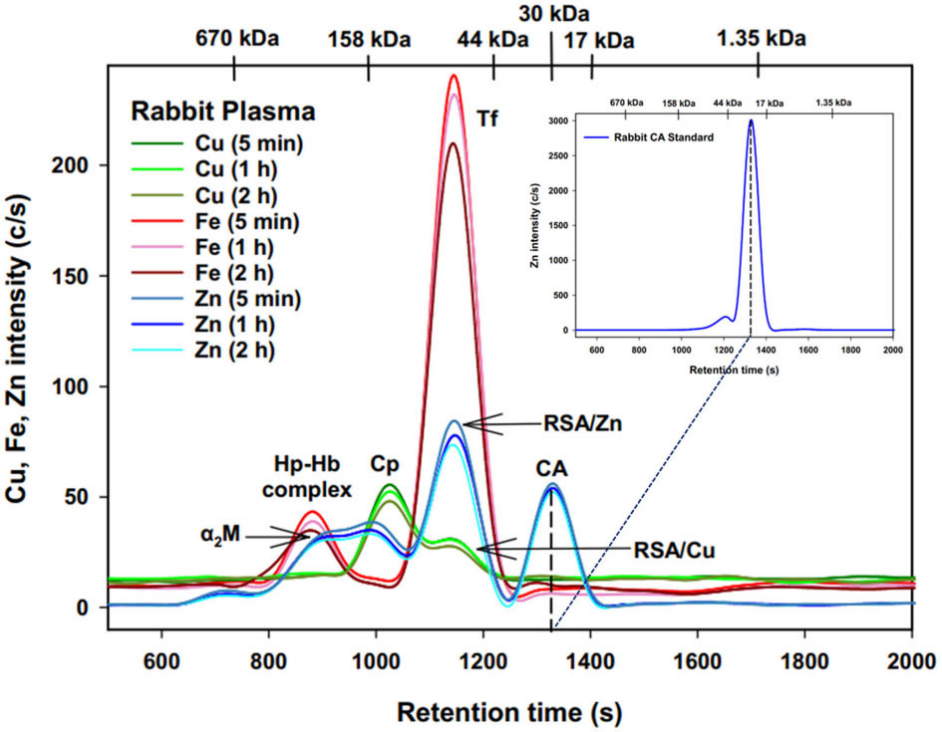
Representative Cu, Fe, and Zn-specific chromatograms obtained by SEC-ICP-AES for the analysis of rabbit blood plasma that had been spiked with bovine CA after 5 min, 1 h, and 2 h. Column: Superdex 200 HR SEC column (30 × 1.0 cm ID, 8.6 µm particle size, fractionation range: 600–10 kDa); Mobile phase: PBS-buffer (pH 7.4); Temperature: 20°C; Flow rate: 0.75 mL/min; Injection volume: 500 µL. The retention times of a mixture of molecular weight standards and that of pure bovine CA (30 kDa) are indicated at the top of the figure.

**Fig. 2:**
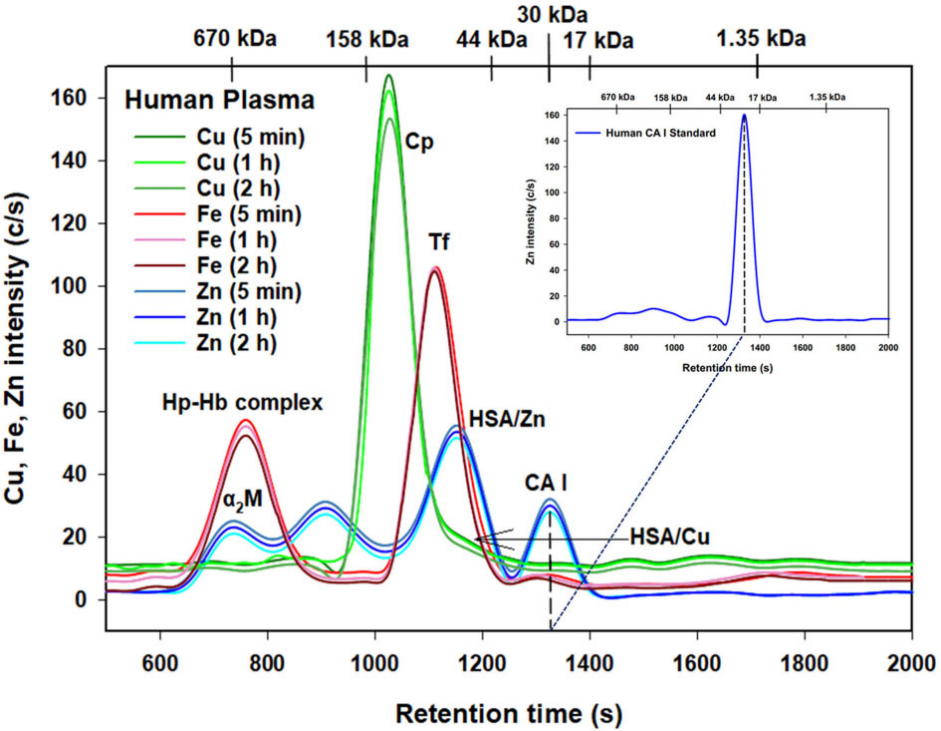
Representative Cu, Fe, and Zn-specific chromatograms obtained by SEC-ICP-AES for the analysis of human blood plasma that had been spiked with human CA I after 5 min, 1 h, and 2 h. Column: Superdex 200 HR SEC column (30 × 1.0 cm ID, 8.6 µm particle size, fractionation range: 600–10 kDa); Mobile phase: PBS-buffer (pH 7.4); Temperature: 20°C; Flow rate: 0.75 mL/min; Injection volume: 500 µL. The retention times of a mixture of molecular weight standards and that of pure human CA I (30 kDa) are indicated at the top of the figure.

The actual experiments involved the temporal SEC-ICP-AES analysis of rabbit plasma that had been spiked with bovine CA (Fig. 1) and human plasma that had been spiked with human CA I over a 2-h time period (Fig. 2). The observed Cu, Fe, and Zn peaks were assigned to their corresponding plasma metalloproteins based on prior investigations conducted with rabbit^19^ and human plasma.^14^ In brief, the observed endogenous metalloproteins correspond to the Cu-proteins ceruloplasmin (Cp) and RSA/HSA-bound Cu (RSA/HSA/Cu), the Zn-proteins α_2_ macroglobulin (α_2_M), RSA/HSA-bound Zn (/RSA/HSA/Zn), and two Fe-proteins, namely the haptoglobin-haemoglobin complex (Hp-Hb) and holo-transferrin (Tf). In accordance with the control experiments, the Cu, Fe, and Zn metalloproteome profiles did not change appreciably over the 2-h time period for both rabbit and human plasma (Figs. 1 and 2). Correspondingly, the total peak areas that were obtained for Cu, Fe, and Zn did also not change appreciably over the 2-h period (Table 1). Most importantly, the total Zn area corresponding to endogenous Zn-proteins in rabbit and human plasma did not change after the addition of CA, and the total Zn area corresponding to the added exogenous CA and CA I also remained constant. These results conclusively demonstrate that CA did not undergo binding to endogenous plasma over the investigated 2-h period (Table 1).

**Table 1.** Total peak areas of all detected peaks based on the Cu, Fe and Zn-specific chromatograms following the analysis of rabbit and human plasma spiked with CA using SEC-ICP-AES at the different time points (*n* = 3).

		Mean total peak area			
		Endogenous Cu	Endogenous Fe	Endogenous Zn	Exogenous Zn (CA)
Rabbit Plasma	Control _5 min_	_	_	17 512 ± 121	_
	*t*_5 min_	5865 ± 34	25 327 ± 221	17 243 ± 103	4667 ± 16
	*t*_1h_	5787 ± 26	25 069 ± 216	16 997 ± 117	4529 ± 21
	*t*_2h_	5691 ± 22	24 835 ± 214	16 465 ± 132	4482 ± 12
Human Plasma	Control _5 min_	_	_	14 167 ± 48	_
	*t*_5 min_	15 501 ± 31	16 637 ± 64	13 952 ± 56	2726 ± 11
	*t*_1h_	15 302 ± 27	16 465 ± 83	13 682 ± 71	2645 ± 9
	*t*_2h_	15 089 ± 62	16 264 ± 108	13 486 ± 87	2575 ± 23

**Fig. 3:**
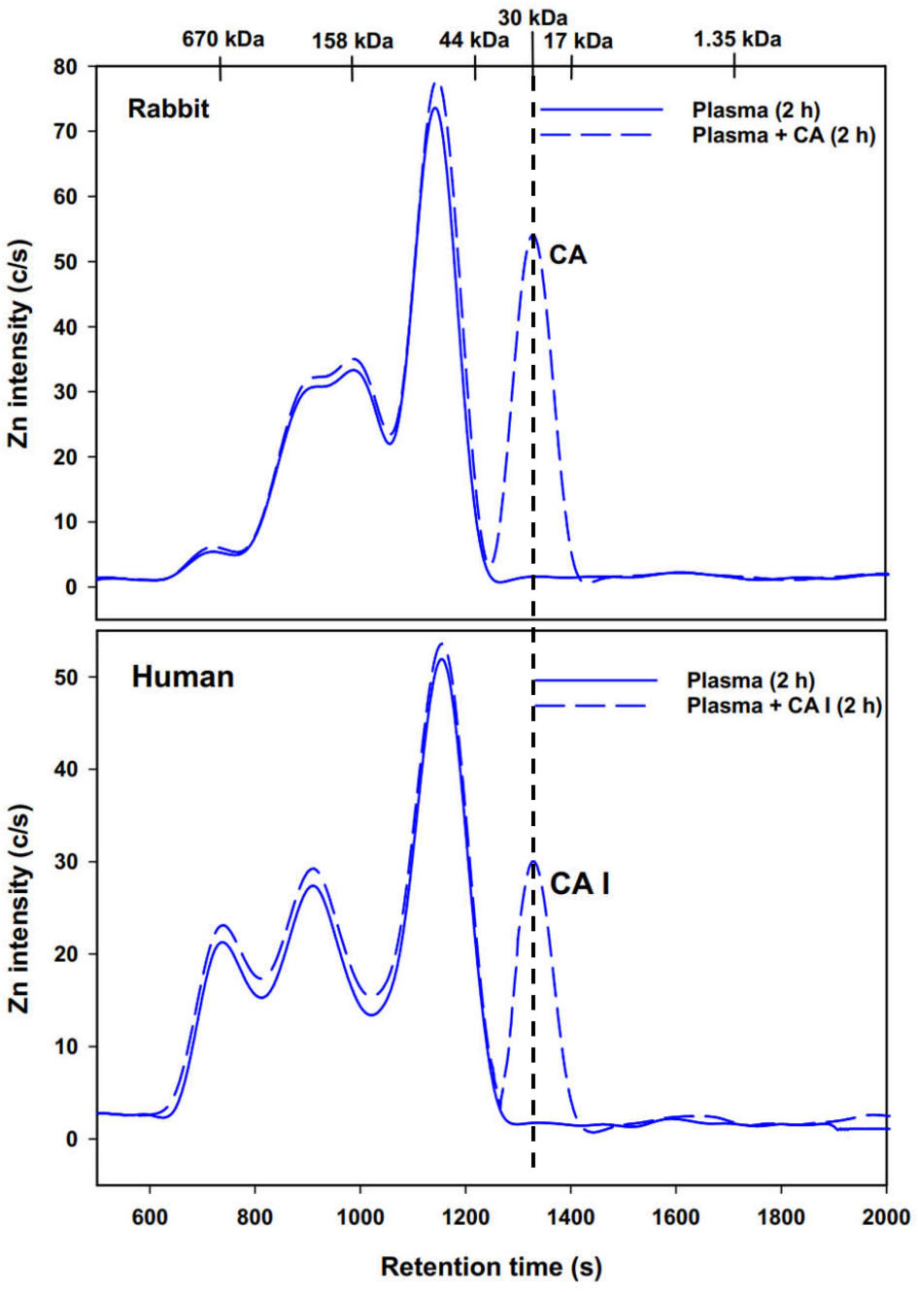
Representative Zn-specific chromatograms obtained by SEC-ICP-AES for the analysis of rabbit blood plasma that was spiked with bovine CA after 2 h (top) and human blood plasma that was spiked with CA I. Column: Superdex 200 HR SEC column (30 × 1.0 cm ID, 8.6 µm particle size, fractionation range: 600–10 kDa); Mobile phase: PBS-buffer (pH 7.4); Temperature: 20°C; Flow rate: 0.75 mL/min; Injection volume: 500 µL. The retention times of a mixture of molecular weight standards and that of pure bovine CA (30 kDa) are indicated at the top of the figure.

Importantly, the simultaneously obtained Zn-specific chromatograms revealed an additional single Zn peak that must correspond to the added bovine CA and human CA I (Figs. 1 and 2, blue lines). This Zn peak was separated from all endogenous Zn metalloproteins and consistently emerged at all investigated time points after HSA-bound Zn, with a retention time corresponding to a 30 kDa protein. The observed high reproducibility of the Zn results over the 2 h period (Figs. 1 and 2, blue lines) implies that neither bovine CA nor human CA I bound to plasma proteins in rabbit or human blood plasma, respectively. To more clearly depict the Zn results obtained for CA and CA I spiked rabbit and human plasma, which are most relevant in the context of our investigations, the corresponding Zn results were overlaid (Fig. 3).

**Fig. 4:**
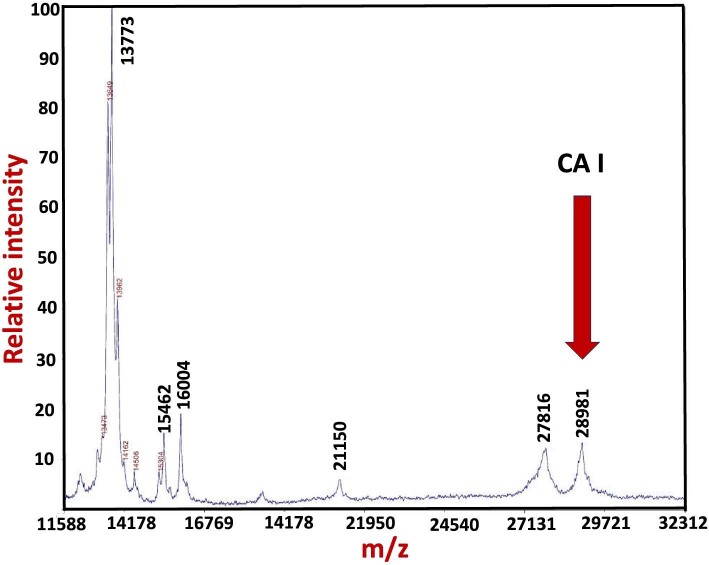
MALDI-TOF MS identification of the Zn-peak as CA I after SEC-ICP-AES analysis using 50 mM Tris buffer (pH 7.4) as the mobile phase.

To corroborate these findings, the experiment involving spiking human plasma with CA I was repeated using a 50 mM Tris buffer mobile phase. Subsequently, the CA I-containing Zn peak was collected and analysed by MALDI-TOF MS (Fig. 4). The results revealed a protein with a m/z ratio of 28,981 Da, closely aligning with the experimental value of 28,958 Da obtained for pure bovine CA. Although the chromatographic resolution of SEC is inherently limited,^29^ the results obtained by SEC-ICP-AES (Fig. 3) and those from analysing the Zn-fraction, which eluted almost baseline separated from all other endogenous Zn-metalloproteins by MALDI-TOF MS (Fig. 4), identify the additional Zn-peak as CA I and therefore conclusively demonstrate the absence of a binding protein for CA I in human plasma in vitro.

Putting these findings into context, few studies have reported on the determination of the concentration of CA I in human blood plasma, with some studies reporting no appreciable concentrations in adults^30^ and male neonates,^31^ while others reported 2.1-fold higher CA I concentrations in blood plasma samples from breast cancer patients compared to healthy controls.^32^ These results suggest that once these Zn-metalloprotein is released into the blood plasma, it is likely to be rapidly cleared from the bloodstream by processes that are presently not well understood. Therefore, the analysis of patients plasma with the employed metallomics technique for this Zn metalloproteins is not useful to diagnose a haemolytic anaemia event in the bloodstream, whereas the detection of Fe-metalloproteins would be highly useful to detect such an event.^7^ Notably, other analytical methods are also available for the diagnosis of haemolytic anaemia, which are similarly based on the detection of released Hb in plasma.^12^

The direct experimental observation that CA I remains essentially ‘free’ after its release from RBCs into plasma (Fig. 2) is reminiscent of the previously observed similar behaviour of the small molecular weight and non-toxic arsenic compound arsenobetaine—which is naturally present in many seafoods—in rabbit and human plasma.^18^ The arsenobetaine results provided a feasible explanation for the rapid urinary excretion of this arsenic compound within ∼7–9 h after the ingestion of an arsenobetaine-containing meal (e.g. a rock lobster).

Conversely, CA is a 30 kDa protein which could have a longer residence time in the bloodstream and therefore conceivably engage in adverse biochemical processes that unfold at the bloodstream-endothelium barrier.^33^ In this context, it is crucial to consider that previous studies have revealed that RBCs tend to undergo rapid lysis in the highly oxidative environment of atherosclerotic plaques.^34^ This suggests that the CA I released together with Hb may have a role in the pathogenesis of atherosclerotic lesions as well as the disease progression, particularly since Zn is recognized to affect lipid metabolism, which is implicated in the disease aetiology.^35^

Further evidence supporting a direct biomolecular mechanism linking the release of CA I from RBCs and atherosclerosis comes from studies that have demonstrated the active involvement of this Zn metalloprotein in the calcification associated with atherosclerosis in the aortas of mice.^36^ Although in these experiments CA I was present within the aortic cells, one cannot exclude the possibility that CA I, when delivered to the aortic atherosclerotic lesions via the bloodstream, may have been internalized into the cells by endocytosis^37^ and contributed to disease progression. Relatedly, it has also been demonstrated that CA I released from RBCs contributes to the development of proliferative diabetic retinopathy and macular oedema, leading to vision loss in working-age adults.^38^ Furthermore, CA I also plays a role in the microcalcification and tumorigenesis in breast cancer patients.^32^ Another human disease that is possibly related to the interaction of CA I at the blood-endothelium barrier is multiple sclerosis, which appears to be associated with the dyshomeostasis of the metabolism of Zn and involves the immune system attacking human tissues.^39^ Last but not least, the fact that CA remains ‘free’ in plasma after its release from RBCs, but has been infrequently detected in human plasma from patients implies that it must be readily translocated to internal organs. Therein, CA may either be catabolized and/or excreted via the kidneys as the intact Zn-metalloprotein in the urine.^40^

## Conclusion

The biomolecular events that unfold after toxic metal(loid) species, viruses, bacteria, as well as parasites enter the mammalian bloodstream are incompletely understood and can involve the rupture of RBCs.^41^ To gain insight into the biomolecular processes that unfold after CA I—the second most abundant metalloprotein contained in RBC cytosol after Hb—is released from ruptured RBCs into plasma, we have employed an established metallomics technique. The inherent capability of this bioanalytical tool to directly observe the fate of CA I in this exceedingly complex biological fluid conclusively revealed that CA I does not appear to bind to any plasma proteins over a 2-h time period *in vitro*. These results are in stark contrast to RBC-derived Hb, which effectively binds to the plasma protein Hp to form Hp-Hb complexes and therefore precludes the influx of highly toxic free Hb into the kidneys, which would otherwise cause kidney damage. The observation that CA I essentially remains ‘free’ in human plasma suggests that this otherwise rather stable Zn-metalloprotein could be involved in adverse biochemical processes that unfold at the bloodstream-endothelial barrier. To this end, it has been reported that CA I is involved in the aetiology and progression of atherosclerosis,^36^ as well as vision loss,^38^ and that CA I is also implicated in the microcalcification and tumorigenesis in breast cancer.^32^ Given the general lack of evidence that CA I is present in human plasma in previous studies, our findings imply that this Zn metalloprotein is rapidly cleared from the bloodstream by yet unknown biochemical processes. Furthermore, our results demonstrate that the employed metallomics technique is eminently suited to systematically investigate the biochemical fate of other stable metalloproteins after they are released either from organ tissues (e.g. based on an organ-based disease process) or from RBCs into the bloodstream. Further studies in this general research direction have the potential to unravel the nature of some of the other endogenous Zn metalloproteins that have been detected in human plasma, but that have not yet been conclusively qualitatively identified.^14^

## Supplementary Material

mfae028_Supplemental_Files

## Data Availability

The data underlying this article will be shared on reasonable request to the corresponding author.
