# Supplementary figures and images for: Interaction of carbonic anhydrase I released from red blood cells with human plasma in vitro
Source: Metallomics. 2024 May 29;16(6):mfae028. doi: 10.1093/mtomcs/mfae028 (PMC11188540; doi:10.1093/mtomcs/mfae028)

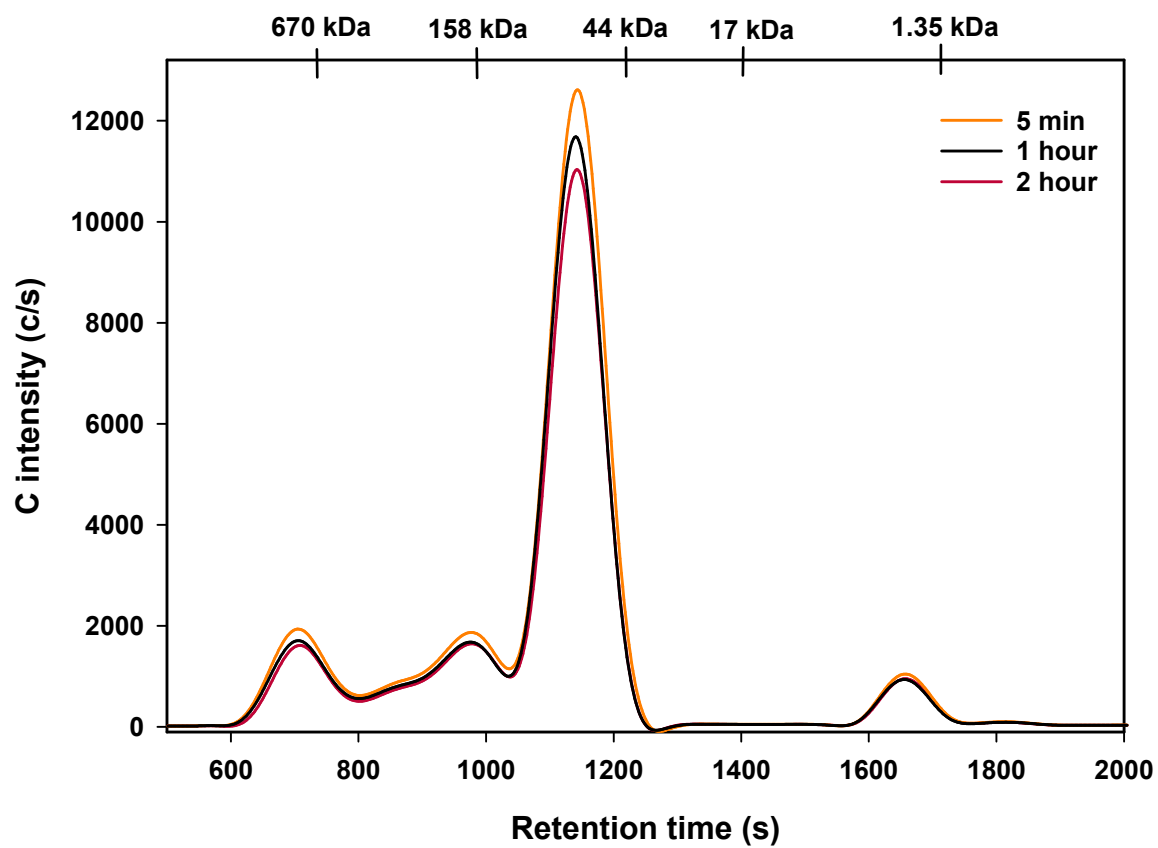

Supplement: mfae028_Supplemental_Files [file mfae028_supplemental_files.zip › Suppl_data_ESI_1.pdf]

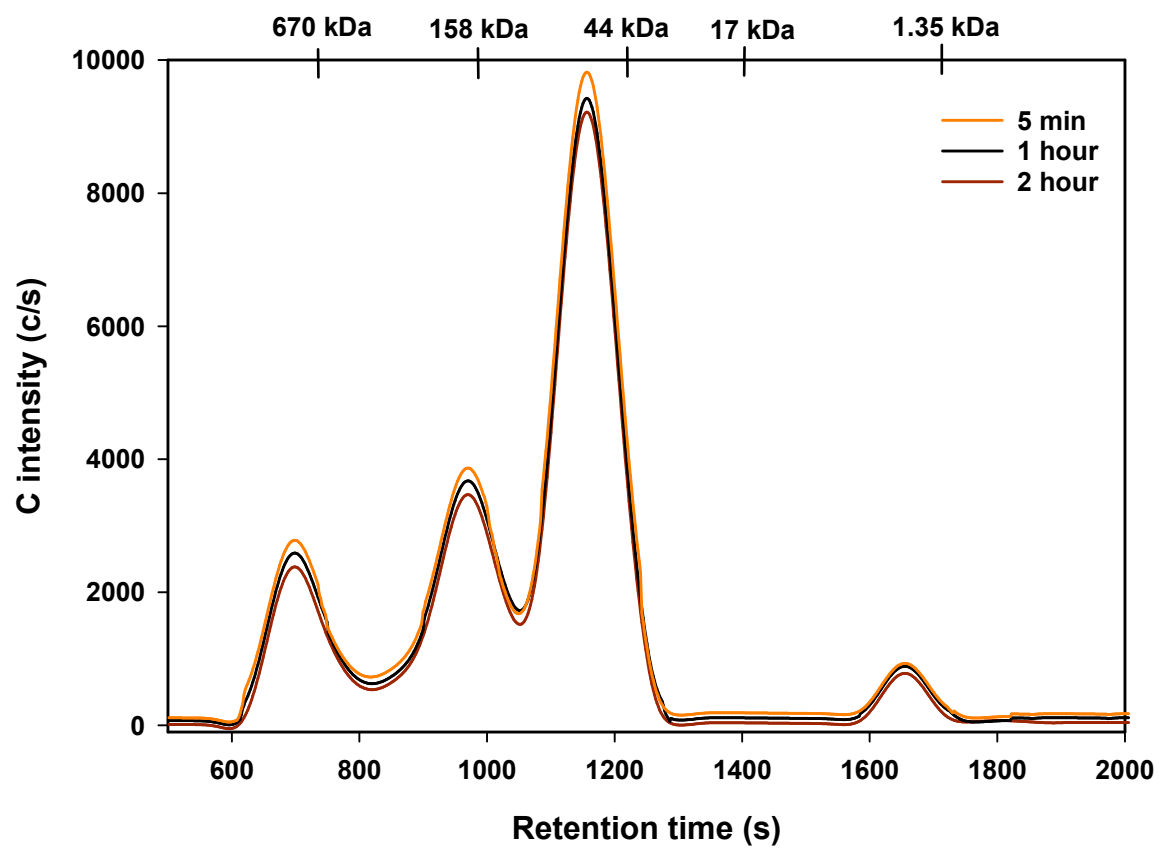

Supplement: mfae028_Supplemental_Files [file mfae028_supplemental_files.zip › Suppl_data_ESI_2.pdf]
